# Fentanyls continue to replace heroin in the drug arena: the cases of ocfentanil and carfentanil

**DOI:** 10.1007/s11419-017-0379-4

**Published:** 2017-08-18

**Authors:** Nektaria Misailidi, Ioannis Papoutsis, Panagiota Nikolaou, Artemisia Dona, Chara Spiliopoulou, Sotiris Athanaselis

**Affiliations:** Department of Forensic Medicine and Toxicology, Faculty of Medicine, National and Kapodistrian University of Athens, 75 Mikras Asias, 115 27 Athens, Greece

**Keywords:** Ocfentanil, Carfentanil, Synthetic opioids, Gray deaths, Toxicology, Legal status

## Abstract

**Purpose:**

Ocfentanil and carfentanil are two potent synthetic opioids that are analogues of fentanyl and are actively involved in the recent fentanyl crisis. The aim of this review is to provide all the available information on these two fentanyl analogues.

**Methods:**

All reviewed information was gathered through a detailed search of PubMed and the World Wide Web using relevant keywords.

**Results:**

Like most of the members of the family of fentanyls, they are either sold as heroin to unsuspecting users or used extensively to lace heroin street samples. Despite the fact that ocfentanil was studied clinically in the early 1990s, it did not manage to find its place in clinical practice. On the other hand, carfentanil is mainly used today as an anesthetic agent in large animals. Ocfentanil and carfentanil are used and abused extensively, mainly in Europe and in the United States. As a result, they are the cause of some verified intoxication cases and deaths worldwide. This review provides information concerning chemistry, synthesis, prevalence, pharmacology, and toxicology, as well as the current legal status of these two fentanyl analogues. Analytical methods developed for the determination of ocfentanil and carfentanil in biological specimens and seized materials, as well as related intoxication and lethal cases are also presented.

**Conclusions:**

Ocfentanil and carfentanil are undeniably very dangerous opioid drugs and a very serious matter of concern for public safety. The authorities should take the appropriate actions to avoid the expansion of this threat by taking proper and prompt measures.

## Introduction

New psychoactive substances (NPS) are a group of substances that are sold in pure form or as a mixture with other drugs of abuse and are widely abused. Most of these substances are not controlled under the 1961 Single Convention on Narcotic Drugs or the 1971 Single Convention on Psychoactive Substances [1]. The rapid spread of NPS in the illegal drug market poses a significant risk for the public health and seems to present a serious matter of concern because of a high number of these drugs emerging every year [2, 3]. Synthetic opioids hold a significant position among NPS as they are highly potent drugs that are often sold as heroin to unsuspected drug users [3]. Among them, fentanyl is an important substance that is used extensively as a powerful pain killer and is also abused by a large number of drug addicts [4, 5]. More than 1400 fentanyl analogues have been synthesized over the years in order to mimic fentanyl’s opioid effects and outsmart the law because fentanyl is a controlled substance in most countries internationally [6, 7]. Two hundred of these analogues have been studied pharmacologically, 12 of which have entered the illegal drug market during the last 5 years [6].

Ocfentanil is a short-acting fentanyl analogue that was firstly described in a US patent by Huang et al. [8]. It had been studied for its analgesic activity and was found to be more potent than fentanyl and with fewer adverse effects, regarding cardiovascular effects and respiratory depression [9]. The drug has also been studied as a supplement in general anesthesia [10]. Ocfentanil was the cause of some verified intoxication cases and deaths, mainly in Europe, and threatens to be an imminent hazard to the public safety [6, 11–15]. Some ocfentanil-related cases have been also presented at different scientific conferences [16–18].

Carfentanil is the most potent of the commercially available fentanyl analogues [19, 20]. It was synthesized in 1974 by a group of chemists at Janssen Pharmaceuticals, and since then it has been used in veterinary medicine as a tranquilizer for the sedation of large animals [21–29]. Carfentanil has been pharmacologically studied mostly in animals, but also in humans [23, 28–39]. Some evidence suggests that it has been used as a chemical weapon by the Russian military, while attempting to subdue a terrorist siege of the Moscow Theater [40, 41]. Carfentanil is often used to adulterate heroin, fentanyl, and other drugs of abuse [19, 42, 43]. During recent years, this fentanyl analogue has been responsible for a significant number of accidental intoxication cases and deaths mostly in the United States [40, 44–46]. Intentional intake of carfentanil does not seem to happen very often, because most drug users are well informed about its potency and its harmful potential [47–49].

The aim of this review is to provide all the available information on ocfentanil and carfentanil, regarding their chemistry, synthesis, prevalence, pharmacology, toxicology, and their current legal status. Analytical methods for the determination of ocfentanil and carfentanil in biological specimens and seized materials, as well as related intoxications and fatal cases are also presented. All the reviewed information was gathered through a detailed search of PubMed and the World Wide Web using the keywords “ocfentanil”, “carfentanil”, “pharmacology”, “toxicology”, “intoxications”, “fatalities”, “determination”, “biological fluids”, and “legal status”.

## Ocfentanil

### Chemistry

**Fig. 1:**
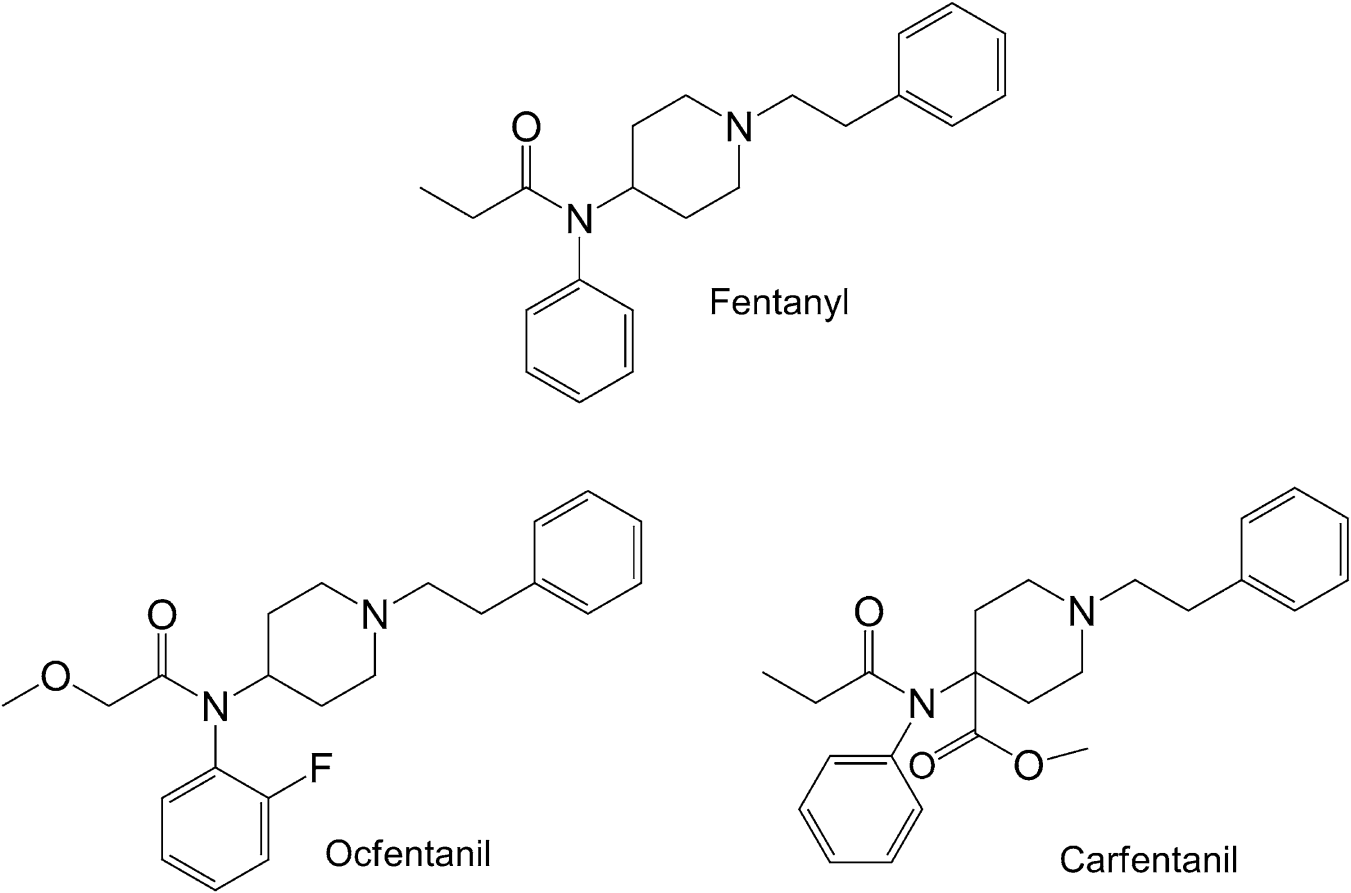
Structures of fentanyl, ocfentanil, and carfentanil

Ocfentanil is a synthetic analogue of fentanyl (Fig. 1) that possesses a methoxy group instead of a methyl group and a fluorine atom on the ortho position of the aniline group [50, 51]. Ocfentanil and ocfentanilum (in Latin) are the proposed international nonproprietary names given by the World Health Organization (WHO) in accordance with article 3 of the Procedure for the Selection of Recommended International Nonproprietary Names for Pharmaceutical Substances [52]. Its International Union of Pure and Applied Chemistry (IUPAC) name is *N*-(2-fluorophenyl)-2-methoxy-*N*-[1-(2-phenylethyl)-piperidin-4-yl] acetamide, but it can also been found under the names 1-(2-phenylethyl)-4-[*N*-(2-fluorophenyl)methoxyacetamido]piperidine hydrochloride, *N*-(2-fluorophenyl)-2-methoxy-*N*-[1-(2-phenylethyl)-4-piperidinyl]acetamide, ocfentanyl, ocfentanilo, UNII-MX52WBC8EV and A-3217 [53]. Ocfentanil is sold either as its base with the molecular formula C_22_H_27_FN_2_O_2_ (molecular weight 370.46 g/mol) or as its hydrochloric acid salt C_22_H_27_FN_2_O_2_ HCl (molecular weight 406.9 g/mol) in a white or brown crystalline powder form [50, 53, 54]. The Chemical Abstracts Service (CAS) number of ocfentanil is 101343-69-5 [53–55]. It has a melting point of 183–184 °C, and there are no data regarding its boiling point [8, 54]. The drug is soluble in aqueous media at a pH below 7 (p*K*_a_ = 7.82) and stable to moderate heat and light, if stored properly [10, 54].

### Synthesis

**Fig. 2:**
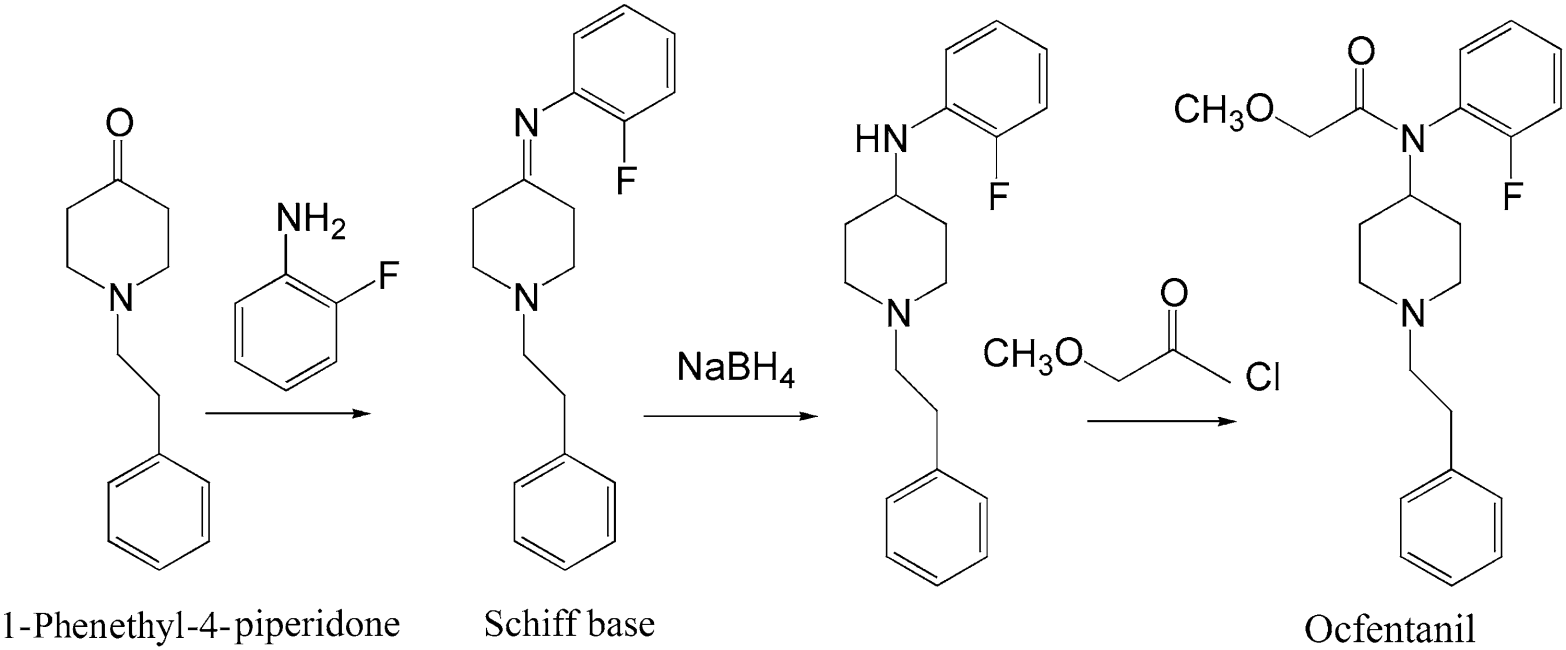
Synthesis of ocfentanil according to Huang et al. [8]

The synthesis of ocfentanil was first described in US patent number 4,584,303 by Huang et al. [8]. Initially 1-phenethyl-4-piperidone reacts with 2-fluoro aniline. The resulting compound is a Schiff base that is reduced to the corresponding diamine, by sodium borohydride or lithium aluminum hydride. The acylation of the secondary amino group with 2-methoxyacetyl chloride (methyl chloroformate) leads to the final product by yielding 1-(2-phenylethyl)-4-[*N*-(2-fluorophenyl)methoxyacetamido]piperidine. The whole synthetic route is presented in Fig. 2 [8, 51].

### Prevalence and use

Despite the fact that ocfentanil had been studied clinically during early 1990s, it did not manage to find its place in clinical practice [10, 56–58]. As a drug of abuse, it emerged recently on the illegal drug market along with other fentanyl analogues [12–14, 59]. Ocfentanil was among the 81 NPS that were reported to the Early Warning System (EWS) of European Monitoring Centre for Drugs and Drug Addiction (EMCDDA) in 2013, after the drug was identified in a seizure made by Dutch police, which might have been intended for sale as “synthetic heroin”, in October 2013. A related alert for ocfentanil was issued by EMCDDA and Europol in the same year [44, 60].

According to the Early Warning Advisory (EWA) of the United Nations Office of Drugs and Crime (UNODC), ocfentanil is among the 14 fentanyl analogues that have been reported in Europe within 2012–2016 and it is among the 15 different synthetic opioids that have been reported to EMCDDA during 2009–2015 [6, 15]. The National Drug EWS (NDEWS) also issued an alert for ocfentanil among other fentanyl analogues [61]. It has been associated with some intoxication cases and deaths mostly in Europe [6, 11–14, 62]. Ocfentanil has also been reported in Finland, France, Ireland, Luxembourg, Spain, Sweden, and the United Kingdom [6, 59, 63–66]. The drug is also among other synthetic opioids that are a current matter of concern in the United States and Canada [67, 68]. Until now it has not been reviewed by the WHO Expert Committee on Drug Dependence (WHO ECDD) [14].

Ocfentanil’s increasing presence in the illegal drug market is undoubtedly pointed out by ocfentanil-related conversations in drug forums and related seizures [12–14, 50, 59, 69–74]. It is seized mostly in white or brown powdered form [12–14, 50, 59, 66, 69–71]. A recent seizure of a pink colored powder has been also reported [17]. Upon analysis of the seized powders, the drug has been found in mixtures with drugs or substances such as heroin, methamphetamine, acetaminophen, caffeine, mannitol, or benzoic acid [12, 13, 50, 59, 65, 69–71]. In many cases, ocfentanil is used to adulterate heroin; thus heroin users can be exposed to ocfentanil without knowing it [12–14, 50, 59, 65, 68–71, 75].

Cases in which fentanyls are sold under the guise of heroin are common worldwide [6]. In some, ocfentanil was labeled and sold in the darknet as heroin or “synthetic heroin” [6, 44, 59, 76]. It was firstly reported in EWS within a seizure of “synthetic heroin” in the Netherlands [44, 50, 70]. Furthermore, four samples, purchased in or sent from France and Spain, were analyzed by Energy Control in Barcelona, two of which were found to contain ocfentanil, caffeine, and acetaminophen. All samples were labeled and sold as heroin on the hidden web [59]. An alert was notified to public and the authorities and was quickly spread throughout the web. The information reproduced in English, French, German, Swedish and Polish drug forums and online discussions pointed out two vendors that were eventually banned from deep web marketplaces [59, 71–74].

The drug users are not always aware of what they purchase and can be unintentionally exposed to unknown substances [12, 13, 65, 76]. Financial profit seems to motivate drug dealers to adulterate heroin with fentanyls such as ocfentanil or to sell them disguised as heroin, as they are presumably cheaper than heroin, although heroin’s price is continuously coming down [65].

The main routes of ocfentanil administration are snorting, injecting, ingesting, and smoking, which are described in ocfentanil-related intoxication cases. The information on the routes of administration of ocfentanil can be found in drug forums [12, 13, 59, 65, 66, 76].

### Pharmacology and toxicology

Ocfentanil was initially synthesized and evaluated for its analgesic activity by Huang et al. [8] in 1986. Its median effective dose (ED_50_) was measured in a mouse hot-plate analgesia test and found to be 0.0077 mg/kg mice [8]. It is estimated that ocfentanil is approximately 2.5 times more potent than fentanyl [10, 12]. Although ocfentanil has been studied for its analgesic potency, there are no available data about its pharmacokinetics.

Ocfentanil was firstly evaluated in humans by Glass et al. [56] in 1989. Analgesic and hemodynamic effects of the drug were studied in this assay. It was administered in eight groups of four volunteers each, at increasing doses. One volunteer from each group was administered placebo, and the other three of the same group received active ocfentanil. Hemodynamic parameters were constantly recorded. Ocfentanil was found to have no significant effect on blood pressure, heart rate, and histamine levels. Its therapeutic dose for humans was calculated to be 0.5–1.25 μg/kg, and it was found to be 200 times more potent than morphine. This dose is more likely to produce analgesia that lasts for 20–40 min [56].

Ocfentanil was observed for its hemodynamic effects on 12 patients with ischemic heart disease. All the patients had a low ejection fraction (the amount of blood pumped out of the ventricle versus the total amount of blood in the ventricle) and no history of myocardial infraction, hemodynamic instability, or recent drug use. The patients were pre-medicated with diazepam and scopolamine, and after stabilization, they received either ocfentanil in increasing doses from 0.5 to 3.0 μg/kg every 10 min (total dose 5 μg/kg), or equal volumes of normal saline. Hemodynamic indices were recorded every 15 min versus placebo (normal saline). Patients eventually experienced a mild decrease of arterial blood pressure. Increased arterial pCO_2_ was also noticed at the time of maximum analgesia and sedation. The experiment concluded that ocfentanil may produce safe analgesia, despite hypercarbia and decreased pulmonary arterial pressure. Patients in the study maintained their hemodynamic performance, and the hemodynamic changes noticed were attributed to secondary drug effects [57].

Later, the pharmaceutical company Anaquest developed and studied ocfentanil among other potent naloxone-reversible opioids in order to gain better therapeutic indices in terms of cardiovascular effects and respiratory depression than those of fentanyl. Ocfentanil was shown to have pharmacodynamic advantages over fentanyl in terms of tendency to accumulate (measured by duration of action), because its ED_50_ was found to be 8–16 times greater than fentanyl’s. Data obtained in rats also suggested a three- to fourfold greater separation between hypnotic and analgesic ED_50_ values for ocfentanil than fentanyl, suggesting a more selective approach to each of these properties [10]. Fletcher et al. [10] designed a study in order to determine the efficacy of ocfentanil and to compare it to fentanyl in terms of potency, when used as supplement in general anesthesia. The study involved 60 adult patients scheduled for elective surgery. The patients were pre-medicated with diazepam, metoclopramide, and ranitidine, and they were randomly administered ocfentanil at different doses from 1 to 5 μg/kg. Hemodynamic variables were recorded 20 min after anesthetic induction for the first 5 min after surgical incision and every 5 min thereafter. The lowest dose of 1 μg/kg of ocfentanil was associated with increased systolic blood pressure and increased heart rate compared to the other doses. The research concluded that ocfentanil did not show any obvious advantage as compared to fentanyl and that a 3 μg/kg dose of ocfentanil was pharmacodynamically equal to a 5 μg/kg dose of fentanyl [10].

Ebrahim et al. [58] evaluated ocfentanil for its safety and effectiveness in postoperative analgesia. Sixty patients were assigned to four groups of 15 each. Three groups received ocfentanil at different doses (0.1, 0.5, 0.75 μg/kg) and the fourth received morphine 0.07 mg/kg. Fentanyl 2.5 μg/kg was used for induction, and isoflurane along with nitrous oxide were used as anesthetic agents. Its patient’s pain was evaluated via Visual Analog Scale for pain (VAS pain) prior to administration and at 5, 10, 15, 30, and 60 min after administration. Hemodynamic variables (blood pressure, electrical activity of the heart, heart rate, respiration,and saturation of O_2_) were recorded during the study. The study found that 0.5–0.75 μg/kg of ocfentanil produced safe and effective analgesia, proportionate to the same produced by 0.07 μg/kg of morphine. Furthermore, analgesia at this dose of ocfentanil lasted up to 1 h in 50% of the patients [58], contrary to what Glass et al. [56] had concluded.

Leslie et al. [57] mentioned that previous animal studies with dogs and rats indicated that ocfentanil may be an effective anesthetic agent in animals. In these studies a decrease of hemodynamic indices (heart rate, mean arterial pressure, and cardiac output) were observed, when ocfentanil was administered at a dose of 18 μg/kg along with isoflurane [57]. In another animal study, ocfentanil was found to have better therapeutic indices in terms of cardiovascular and respiratory effects than fentanyl. Both opioids were administered at equal doses in rats, during rat tail-flick and hot-plate tests. Ocfentanil displayed shorter action as compared to fentanyl in both tests. Therapeutic indices were measured for depression of mean arterial blood pressure, heart rate, and respiration in a group of isoflurane-anesthetized rats. Depression of respiration was also measured in conscious, freely moving rats. In all cases, ocfentanil was found to have better therapeutic indices, while at the same time the vital physiological responses remained in normal levels. Collectively, ocfentanil was concluded to be a potent analgesic drug that could be used in surgery without coadministration of a general anesthetic [9]. Nevertheless, data from animal studies are difficult to get aligned with data from humans, because animals differ significantly in sensitivity and pharmacodynamic effects, and their analgesia is differently accessed from that of humans [10].

Ocfentanil shows typical opiate-like effects like euphoria and relaxation. Users reported that it is less “cool” and euphoric than heroin, but it is more stimulant and has a very quick onset, after 3 min. The users also reported that it has shorter duration of action (3 h) than heroin; the withdrawal symptoms appear earlier [9, 10, 56, 57, 59]. Adverse effects of ocfentanil are similar to those of fentanyl and include itching, nausea, and potentially serious dose-related respiratory depression [10, 12, 14, 55, 59, 65, 66, 69]. Other reported adverse effects are chest pain, psychosis, and agitation, although these effects could be due to other coadministered substances [14, 59, 65, 66]. These adverse effects can be harmful and are more likely to depend on the way it is administered rather than on the drug itself [9, 10, 56–59].

### Intoxications and fatal cases

The increased prevalence of ocfentanil worldwide and the trend of labeling ocfentanil as heroin leads to intoxication cases and deaths due to its abuse [6, 14, 44, 50, 59, 67, 70, 76]. Two lethal intoxication cases with ocfentanil have been reported in Belgium [15] and one in Switzerland [12]. More ocfentanil related deaths have been also reported in Ireland and France [6, 17, 18]. Ocfentanil is also involved in intoxication cases and deaths in the United States and Canada, where it is also a concern, among other fentanyl analogues [67]. These deaths show the risks from its use and abuse, and this is the reason why it is already regulated in several countries around the world, especially in Europe [6, 60, 63, 64, 77–89].

EMCDDA and Europol issued another ocfentanil-related alert in 2015, when a death was reported in Belgium, on March 22, 2015 [11]. A 17-year-old man with a history of drug abuse was found dead in his apartment. He was previously hospitalized after using cocaine combined with sleeping pills. The victim was also receiving antidepressant medication until 3 months before the incident. Drug paraphernalia and a small reclosable zipper bag with 2.07 g of a brown powder were found next to the body. Police investigation revealed that the drug had been purchased online with bitcoins and snorted with a straw. The analysis of the powder by high performance liquid chromatography–photodiode array (HPLC–PDA) and gas chromatography–mass spectrometry (GC–MS) revealed the presence of 2.54% ocfentanil along with 58.4% acetaminophen, 13.7% caffeine, and benzoic acid. Liquid-liquid extraction (LLE) and ultra performance liquid chromatography (UPLC) were used to identify and quantify the drug in the postmortem fluids of the deceased. Concentrations of ocfentanil were found to be 15.3 μg/L in femoral blood, 12.5 μg/L in vitreous humor, 23.3 μg/L in cardiac blood preserved with ethylenediaminetetraacetic acid (EDTA) and 21.9 μg/L in cardiac blood without EDTA, 6.0 μg/L in urine, 17.1 μg/L in stomach contents, 31.2 μg/kg in the liver, 51.2 μg/kg in the kidney, 37.9 μg/kg in brain tissue, and finally 13.7 μg/L in bile. Hair analysis revealed that the victim was also a cocaine and heroin user. According to the results of the toxicological analysis, the cause and the manner of death were determined as acute, accidental ocfentanil intoxication [13].

Another case occurred in Switzerland, where a 24-year-old man was found dead in his apartment after consuming a powder that was found to be ocfentanil. The man was known to be a cannabis user, while there was no other information about taking other drugs. A reclosable plastic zipper bag was found close to the deceased containing 405.1 mg of a brown powder. The police investigating the case found that the victim had visited, through his computer, a darknet website and that the powder was delivered from Belgium. No further information could be obtained regarding what he might have ordered. The powder was analysed by GC–MS and revealed the presence of ocfentanil along with acetaminophen, caffeine, and mannitol. Analysis of the powder showed that the ocfentanil content was 0.91%. Postmortem biological samples of the deceased were also analyzed by liquid chromatography–tandem mass spectrometry (LC–MS/MS), and ocfentanil was identified and quantified in them along with citalopram, quetiapine, delta-9-tetrahydrocannabinol (THC), 11-nor-9-carboxy-delta-9-tetrahydrocannabinol (THC-COOH), and 11-hydroxy-delta-9-tetrahydrocannabinol (11-hydroxy-THC). The concentrations of ocfentanil were found to be 9.1 μg/L in fluoride stabilized femoral whole blood, 7.5 μg/L in heparin stabilized femoral whole blood, 27.9 μg/L in cardiac whole blood, 480 μg/L in urine, and finally 360 ng in the nasal swab. Quantification of the other substances yielded 130 μg/L citalopram, <10 μg/L quetiapine, <5 μg/L THC-COOH, and 11-hydroxy-THC under the limit of quantification. The police concluded that the deceased man had snorted the powder and his death was attributed to an acute ocfentanil intoxication [12].

In Dublin and Cork, Ireland, five deaths were attributed to fluorofentanyl and ocfentanil between April and July 2016. The five deaths were further investigated and it seems that the users had either smoked or injected these fentanyl analogues. The drugs were probably sold as mixtures with either heroin or caffeine and acetaminophen. Because of these cases, the Health Service Executive of Ireland issued an alert concerning fentanyl and its analogues in July 2016 [6, 62].

Ocfentanil was reported in April 2017 as the cause of three nonfatal intoxication cases and two deaths in France [17, 18]. The first death concerned a 30-year-old man who was found dead in his apartment next to drug syringes and related paraphernalia. A pink-colored powder was also found near to the body. An autopsy took place and revealed multiple injection sites in the left foot and right elbow, and there were signs of asphyxia. The powder was analyzed by GC–MS, and the results showed the presence of 0.6% heroin, 37% acetaminophen, 44% caffeine, 1.2% ocfentanil, and heroin impurities such as papaverine and noscapine. Biological specimens (cardiac and peripheral blood, vitreous humor, bile, gastric content, hair, and nasal swab) of the deceased were collected and analyzed by LC–MS/MS. The analysis revealed the presence of acetaminophen and caffeine in the blood at concentrations of 3 and 0.7 mg/L, respectively, as well as non-quantifiable traces of morphine (<5 μg/L). Ocfentanil was found in all biological samples at the following concentrations: 3.7 μg/L in the peripheral blood, 3.9 μg/L in the cardiac blood, 2.0 μg/L in the vitreous humor, 8.4 μg/L in the bile, and 2.5 μg/L in the gastric contents. A nasal swab was negative for ocfentanil. Blood alcohol was also determined at a concentration of 0.20 g/L. His death was attributed to acute ocfentanil intoxication [17]. The second fatal case concerned a 29-year-old man who was found comatose in a hotel toilet and died after hospitalization. Traces of powder were found near the body. Screening of the powder was performed by GC–MS, HPLC–DAD, and LC–MS/MS. Ocfentanil was detected in the powder in non-quantifiable traces, along with caffeine and acetaminophen. Blood samples were analyzed by GC–MS. Cocaine (<5 μg/L), THC (1.3 μg/L), THC-COOH (27.8 μg/L), benzoylecgonine (927 μg/L), ecgonine methyl ester (54.5 μg/L), 3,4-methylenedioxymethamphetamine (30.3 μg/L), 3,4-methylenedioxyamphetamine (5.2 μg/L), ephedrine (hospital administered), caffeine, and acetaminophen were detected. Ocfentanil was also found in blood at a concentration of 5.3 μg/L. This concentration could not be evaluated due to the long time interval between the intake of the drug and the blood sampling. Death was not attributed to ocfentanil due to the simultaneous consumption of other drugs [18]. Ocfentanil was also involved in three nonfatal intoxication cases in France. The victims had sniffed the content of capsules that had been bought on a darknet website and passed out almost immediately. One recovered during his admission to the hospital, and the other two during the hospitalization after a coma of 2 h. The seized capsules were analyzed by GC–MS, HPLC–DAD and LC–MS/MS. Ocfentanil (17%) along with caffeine (16%), acetaminophen (51%), and mannitol were determined to be present in the capsules. GC–MS, HPLC–DAD, and LC–MS/MS were used to identify ocfentanil in biological fluids, while its quantification in blood samples was performed by GC–MS. In the first case, ocfentanil (35.2 μg/L) and acetaminophen were found in blood, and ocfentanil (3.5 mg/L), acetaminophen, and amphetamine (21.8 μg/L) were found in urine. In the second case, 13.9 μg/L of ocfentanil was found in blood along with acetaminophen. In the third case, the blood concentration of ocfentanil was found to be 20.7 μg/L, and acetaminophen was also detected. All victims survived after prompt and proper treatment [18]. It has to be mentioned here that the concentrations of ocfentanil in nonfatal intoxications were much higher than those reported by Coopman et al. [13] and Dussy et al. [12], who reported fatal cases.

### Analysis of ocfentanil in seized materials and biological specimens

A screening test based on enzyme-linked immunosorbent assay (ELISA) principles was developed recently by Randox Laboratories Ltd. for “Designed fentanyl and opioids” and includes ocfentanil in an NPS panel along with norfentanyl, carfentanil, remifentanil, and sufentanyl [90].

Chromatographic methods for determining ocfentanil in biological specimens have been developed within the frame of the toxicological investigation of ocfentanil-related cases [12, 13, 17, 18].

A UPLC–MS/MS method was developed by Coopman et al. [13] for determining ocfentanil in biological samples during the investigation of a fatal intoxication case in Belgium. To our knowledge, this is the only published analytical method that includes validation data. Multiple biological matrices were submitted for toxicological analysis (femoral and cardiac blood, vitreous humor, urine, stomach content, liver, kidney, brain tissue, and bile). LLE with a mixture of *n*-hexane/ethyl acetate (7:3, v/v) was used for extracting all biological specimens, while the tissues were homogenized prior to their extraction. Fentanyl-*d*_5_ was used as internal standard (IS). The UPLC–MS/MS system was set in gradient mode, and chromatographic separation was achieved through an Acquity UPLC HSS C18 column and an HSS C18 Vanguard column as a guard column. Formic acid in water (0.15%) and formic acid in acetonitrile (0.15%) were used as mobile phases A and B, and the flow rate was 0.4 mL/min. Ocfentanil was determined in all biological specimens along with other drugs [13]. The brown powder found near the body was also analyzed according to an HPLC–PDA and a GC–MS method previously published by Coopman and Coordonier [91] for identifying active ingredients in counterfeit drugs and pharmaceutical preparations seized from the black market for bodybuilders [91]. The HPLC–PDA analysis of the powder showed the presence of caffeine, acetaminophen, benzoic acid, and an unknown substance that was identified as ocfentanil by a GC–MS system via library research [13].

Dussy et al. [12] modified an LC–MS/MS and an HPLC–PDA method that was developed and validated for determining benzodiazepines and their metabolites in whole blood and serum [92]. This slightly modified method was used to analyze blood and urine samples taken from a fatal intoxication case. Citalopram and quetiapine were initially detected in urine during a GC–MS general unknown screening, while ocfentanil was identified by an LC–MS/MS method. Quantification of the analytes detected in urine was performed in the peripheral blood using the same LC–MS/MS method and fentanyl as IS. The samples were extracted with *n*-chlorobutane and centrifuged. For quantifying ocfentanil in blood and urine, a standard addition procedure was applied after external calibration. The brown powder found nearby the body was dissolved in methanol, filtered, and analyzed by GC–MS. A second GC–MS analysis of the powder took place under the same conditions, after acetylation with acetic anhydride in pyridine environment. The quantification of ocfentanil in the powder was performed by the same LC–MS/MS method that was used for the toxicological analysis of the biological samples [12].

An LC–MS/MS method (positive ionization mode) was used to identify and quantify ocfentanil in biological samples during the investigation of a fatal intoxication case in France. All samples were pretreated with sulfosalicylic acid containing fentanyl-*d*_5_ as IS. Then, the samples were centrifuged and the supernatant was subjected to solid-phase extraction (SPE) through Oasis HLB^®^ C18 columns. The chromatographic separation was performed by using an XSelect^®^ column [17]. In another series of fatal and nonfatal intoxication cases in France, a GC–MS/MS method was used for the determination of ocfentanil in urine and blood, after extraction with De-Tox Tubes A^®^ [18].

### Legal status

Ocfentanil appeared on the illegal drug market during recent years. It is a fentanyl analogue used alone as a substitute or adulterant of heroin. In many cases, it is sold as “synthetic heroin” to unsuspecting drug users. It is responsible for some fatal and nonfatal intoxication cases. This has put the drug under surveillance by WHO as it constitutes an imminent hazard to public safety [14]. Although some ocfentanil-related intoxication cases have been reported to the EWS of EMCDDA, the drug is not generally controlled in the United Nations or European Union, but it is already considered illegal in some countries in Europe and Asia, as well as in the United States [6, 60, 63, 64, 77–89].

Finland defined ocfentanil as a narcotic substance according to the Narcotics Act (373/2008) in July 2015 based on its structure and pharmacological properties. Its manufacture, trading, and possession imply legal penalties; the drug was included in an amendment of Annex IV of the Government Decree [64]. In Estonia, ocfentanil was placed onto the List of Annex 1 to the Regulation No 73 of May 2005 in order to reduce the distribution and abuse of ocfentanil along with five other NPS in July 2015; information from Estonian Forensic Science Institute and data obtained from EMCDDA had been taken into consideration for the amendment of this regulation [77]. This fentanyl analogue was included in the Neptune Guidance of March 2015, as a controlled NPS in the United Kingdom; the drug had been already considered scheduled under the Misuse of Drugs Regulations 2001 No 3998 that came into force on February 1, 2002 [60, 83]. Ocfentanil is also controlled as a Class A substance in Wales [81, 82]. In Sweden, ocfentanil was classified as an illicit narcotic substance among other synthetic opioids in August 2015 [78]. According to the Republic of Lithuania Minister of Health Order No V-1062, ocfentanil was put in the list of narcotic and psychotropic substances, in September 2015 [79]. Οcfentanil was added to the Annex 4 of the Czech Government Regulation in January 2016, as it has no industrial and therapeutic use and it belongs in the family of synthetic opioids. Its scheduling was based on a decision of EWS working group and on reports of it on eight European countries, as there were no reports of ocfentanil in Czech Republic [63]. In Denmark, the Ministry of Health issued the placement of ocfentanil in Annex 1 of the Danish List of euphoriant substances in November 2016 along with eleven other substances [80]. In Italy, ocfentanil was included into Table 1 of the Ministerial Decree 309/90 in August 2016 [86].

In October 2015, the Chinese Food and Drug Administration placed ocfentanil under national control, as a psychoactive substance [6, 87]. In Japan, ocfentanil was designated as “Specified Drug” by the Ministry of Health, Labour, and Welfare, in November 2016; its manufacture, importation, sale, possession, and further ocfentanil-related activities imply legal penalties [88, 89].

In the United States, ocfentanil was put on the Pennsylvania’s list of Schedule I controlled substances as a fentanyl derivative, according to the 2016–2037 Act, which became effective on August 8, 2016 [84]. In the state of Florida, the drug is listed in Schedule I, as it has a high potential of abuse and no current medical use [85].

## Carfentanil

### Chemistry

Carfentanil is a synthetic opioid (Fig. 1) that is an analogue of fentanyl (a carboxylated fentanyl). Its IUPAC name is 4-[(1-oxopropyl)-phenylamino]-1-(2-phenylethyl)-4-piperidinecarboxylic acid methyl ester, but it is also marketed under the names carfentanyl, carfentanila, carfentanilum, Wildnil, UNII-LA9DTA2L8F and 59708-52-0. Other Medical Subject Headings synonyms include 4-carbomethoxyfentanyl, carfentanyl, (4-methoxycarbonyl)fentanyl, ^11^C-carfentanil, carfentanil citrate, carfentanil oxalate, carfentanil (+−) isomer, R31833 and R33799 [23, 93, 94]. It is sold either as its base with the molecular formula C_24_H_30_N_2_O_3_ (molecular weight 394.515 g/mol) or as its citrate salt with the molecular formula C_30_H_38_N_2_O_10_ (molecular weight 586.638 g/mol) [93, 95, 96]. The CAS number of carfentanil is 59708-52-0, and there are no data concerning its boiling and melting points [93, 94, 97]. Carfentanil base is a white powder and has also been identified in seized white, pink, and brown powders along with other substances [43, 46, 98–101]. Carfentanil citrate is highly water soluble with no distinguishing odor [45]. Carfentanil can be structurally confirmed by means of infrared spectroscopy, nuclear magnetic resonance, GC–MS, and isotope ratio mass spectrometry [101].

### Synthesis

**Fig. 3:**
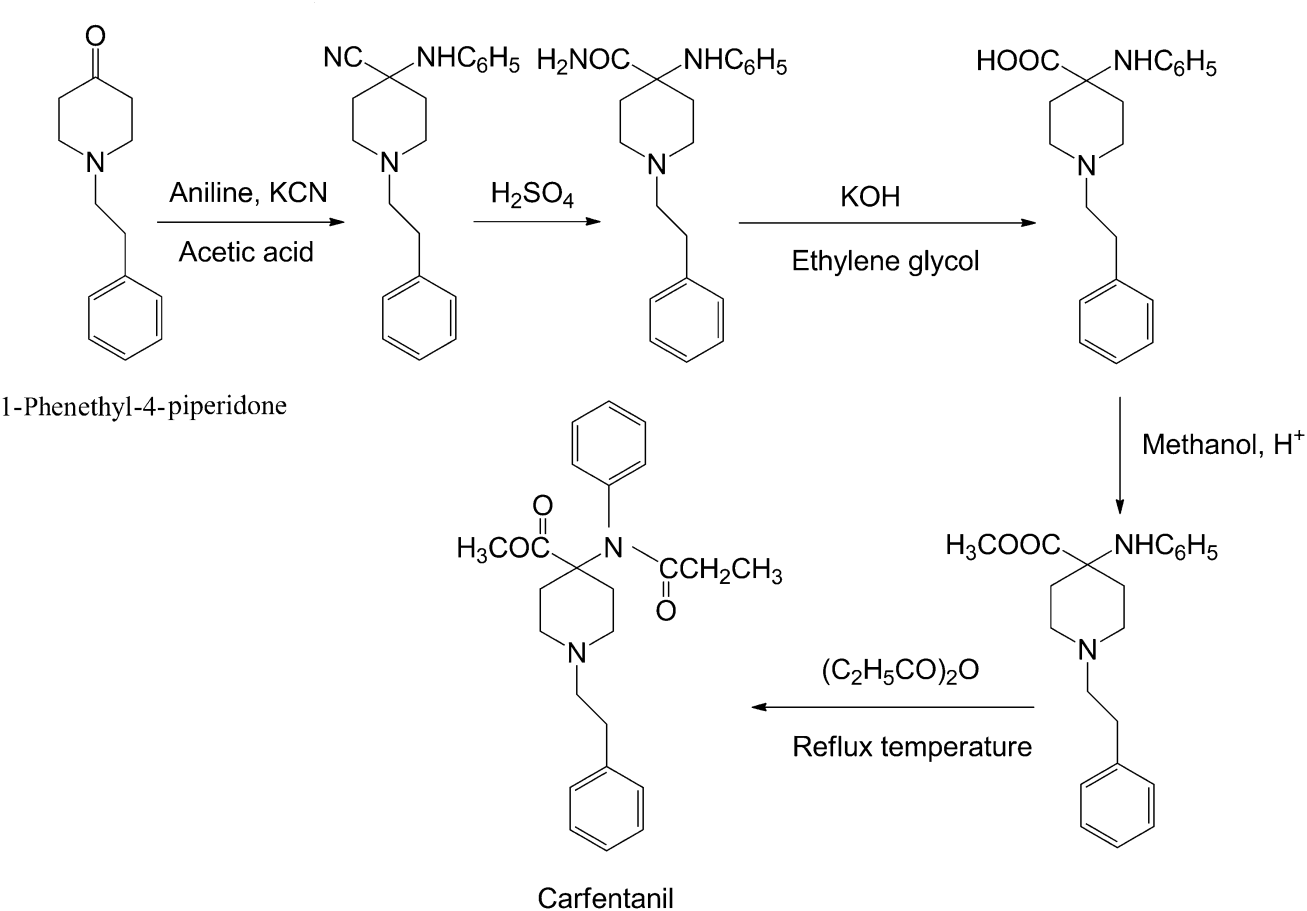
Synthesis of carfentanil according to Janssen and Van Daele [22]

Carfentanil was synthesized by a team of chemists, including Paul Janssen, at Janssen Pharmaceuticals in 1974 [102]. Its synthesis was firstly described in the literature by Van Bever et al. [21] in 1976 and was patented by Janssen et al. (US patent No. 4,179,569) in 1979 [22]. Several synthesis routes for carfentanil have been described in the literature [21, 22, 103–106]. The first developed synthetic procedure starts with the reaction of 1-phenylethyl-4-piperidine with KCN and aniline in acetic acid. The resulting compound is hydrolyzed with cool H_2_SO_4_ and gives the corresponding diamine which is further hydrolyzed with KOH in refluxing ethylene glycol. The acid reacts with methanol under acidic conditions and the resulting methyl ester is acylated with propionic anhydride yielding carfentanil (Fig. 3) [21, 22, 103].

Labeled carfentanil have been also synthesized in order to be used mainly in pharmacology studies on the µ opioid receptor (mOR) binding [104, 105]. A simple synthesis of [^11^C] carfentanil was described by Jewett in 2001. Initially the tetrabutylammonium salt of 4-[*N*-(1-oxopropyl)-*N*-phenylamino]-1-(2-phenylethyl)-4-piperidinecarboxylic acid reacted with [^11^C] methyl triflate in dimethyl sulfoxide (DMSO). The resulting [^11^C] carfentanil is extracted via an Empore SPE extraction disc and all radioactive contaminants are removed. The product is then eluted by a mixture of ethanol and water and passed through an anion exchange column to remove any remains of contaminants [105].

Carfentanil can be also synthesized via the Siegfried route in which 1-phenethyl-4-piperidone is used as a precursor [107], but it is slightly more complex than fentanyl’s [108], because it requires the introduction of a carbomethoxy group [107].

### Prevalence and use

Carfentanil, the active ingredient of Wildnil, is a fentanyl analogue that is approved for veterinary use as a tranquilizing agent for sedation, as a hypnotic, and as anesthesia of animals such as elephants, gazelles, goats, horses, pigs, polar bears, rhinoceroses, seals, and wolves [23–29]. It has been extensively studied in animals since it was first synthesized [23, 28–30, 32, 37, 38, 109], but a few human studies have been also reported [31, 33, 34]. Carfentanil has been characterized as the most potent, dangerous, and commercially available fentanyl analogue. It has been also used as a chemical weapon, making it a very serious hazard to public safety [19, 20, 45]. It has been reported through seizures, intoxication cases, and illicit drug trafficking in Europe, Asia, the USA, and Australia [14, 40, 41, 43, 45, 46, 110–113].

Most of fentanyl analogues including carfentanil are usually manufactured in China and exported from there to all over the world [19, 20, 99, 113, 114]. In October 2016, Associated Press news reported finding 12 Chinese laboratories willing to export carfentanil to the United States, Canada, the United Kingdom, France, Germany, Belgium, and Australia for the price of US$2750/kg [20, 114]. Until March of 2017, carfentanil was not regulated in China; it was openly and legally manufactured and sold by Chinese companies [20, 94]. The drug can be also found easily online, on the darknet, through related websites in which it is often labeled as a “research chemical” and sold through direct mail shipments in prices from US$800 to 2500 per gram [19, 20, 111, 113]. More specifically in 2016, one darknet search engine gave 118 websites selling carfentanil [115]. Because carfentanil is more potent than heroin, its trafficking quantities are significantly less than those of heroin. Therefore, it is easier and cheaper to be smuggled without necessarily being cheap to manufacture [19, 116]. Carfentanil arrives from China in powdered and tablet form, but it also comes in many other forms such as blotter papers, patches, and sprays. In some cases, it has been accidentally absorbed through the skin, inhaled, or ingested [45, 117–119].

Carfentanil has not been reviewed up to now by the WHO ECDD [14]. It was reported for the first time in EMCDDA in February 2013, when it was identified in a seized powder by the Latvian police. In the same period, the Latvian National Local Point issued an alert on carfentanil, which noted that the drug was related to several unconfirmed deaths throughout the country [44].

On September 22, 2016, the Drug Enforcement Administration (DEA) issued a worldwide warning to the public and law enforcement agencies about the risks and hazards of carfentanil, based on the increasing number of carfentanil-related deaths throughout the United States [120]. Carfentanil has been identified across eight states in more than 400 seized materials from July to October 2016 [121]. Most of these seizures were located in Ohio [115]. The DEA recorded prevalence of the drug in a number of states including Florida, Georgia, Rhode Island, Indiana, Pennsylvania, Kentucky, West Virginia, New Jersey, and Illinois, while an intoxication outbreak linked to carfentanil was recorded in July 2016 in Cincinnati, OH [19, 43, 111]. The drug possibly arrived in Ohio through Canada and Mexico, as transshipment points [19, 113, 122]. Since the DEA’s report, carfentanil-related deaths have been also recorded in Florida, Illinois, Colorado, Wisconsin, Minnesota, Michigan, West Virginia, New Hampshire, Virginia, and Maryland, but also some unconfirmed cases have been reported in some other states [119].

In Australia, carfentanil was noted for the first time within a seizure in Sydney in December 2016, where it was identified in a package at a Sydney mail center. Australian police issued an alert in February 2017, when the drug was identified in a package at a mail center in Brisbane [114, 116, 123, 124].

Carfentanil is often used to adulterate heroin, cocaine, and fentanyl. In some other cases, it is labeled and sold as heroin [19, 42]. In Cincinnati, OH, a new drug appeared in 2016 under the street name of “gray death”. It looks like cement and often contains cocaine, heroin, fentanyl, carfentanil, furanylfentanyl, and acrylfentanyl [125, 126]. Carfentanil has also been identified in mixtures along with caffeine, antihistamines, furanylfentanyl, or acrylfentanyl, while in some other cases it has been found to be laced with ketamine [113, 126]. Unconfirmed reports of marijuana laced with carfentanil have been found in northeast Ohio and Canada [125]. Furthermore, the drug has been found in counterfeit pills. The DEA reported that carfentanil had shown up in counterfeit prescription pills sold as OxyContin and Xanax [125, 127].

In some cases of opioid use, the users developed a tolerance to the drug, and they began to chase opioids offering a more intense outcome. However, in the case of carfentanil, more than any other fentanyl analogue, users do not know that its intensified effect can kill them [42]. A user mentioned that he tried a nasal spray of carfentanil [48], while in another drug forum a user sought information about making a carfentanil solution, which was probably intended to be used intravenously [49]. However, many drug users commented: “Never use carfentanil. If someone vends it, he should be banned, because it’s poison” or “Warning: using carfentanil is stupid and deadly and no one should ever do it. Ever” [47]. These comments indicate that opioid addicts are also alerted regarding the harmful potential of this drug.

### Metabolism

**Fig. 4:**
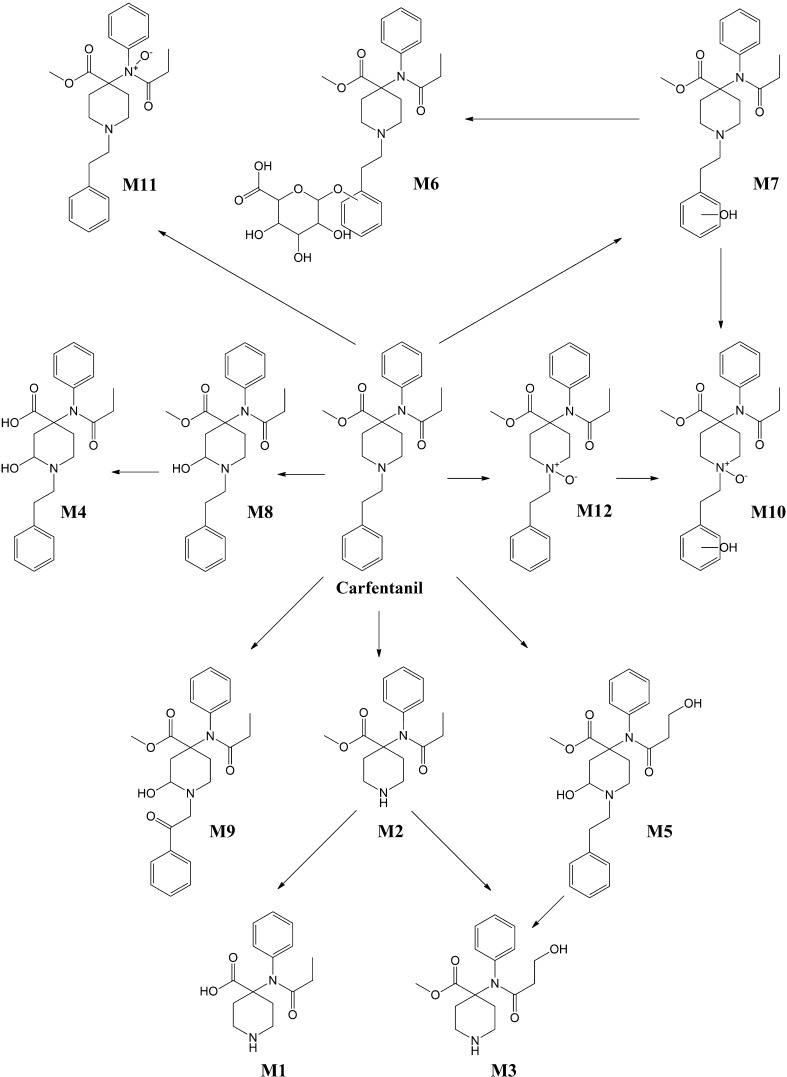
In vitro metabolic pathways for carfentanil in human hepatocytes proposed by Feasel et al. [128]

To the best of our knowledge, there is only one published metabolic study of carfentanil. Feasel et al. [128] described the possible metabolic pathways of carfentanil in humans for the first time in 2016. Two prognostic models, MetaSite software (Molecular Discovery, Pinner, UK) and ADMET Predictor (Simulations Plus Inc., CA, USA), were applied to predict possible in silico carfentanil metabolites. Initially carfentanil (5 μmol/L) was incubated with human liver microsomes (HLM) in order to determine carfentanil’s clearance and to assess its possible toxicity due to its slow metabolism. The HLM samples were treated properly and analyzed via an HPLC system. The HLM half-life was calculated by observation of carfentanil’s depletion over 1 h. The study concluded that carfentanil is readily metabolized by the CYP enzymes; its long in vivo half-life was attributed to other factors. They suggested that the drug’s high lipophilicity and larger volume of distribution makes it less available for hepatic metabolism than its less potent fentanyl analogues. Evidence from other fentanyl analogues suggests that carfentanil probably binds to plasma proteins strongly. They conducted further studies to identify the metabolites of carfentanil after incubation with human hepatocytes. An HPLC system coupled with a triple time-of-flight (TOF) mass spectrometer was used for identification of the metabolites and the analysis was performed under the same chromatographic conditions (column and mobile phases) as with the HLM analysis. Twelve phase I and phase II metabolites were identified through the following metabolic pathways: *N*-dealkylation and ester hydrolysis (M1), *N*-dealkylation of piperidine ring (M2), *N*-dealkylation of piperidine ring and hydroxylation of propanoic group (M3), ester hydrolysis and hydroxylation of piperidine group (M4), hydroxylation of propanoic group (M5), hydroxylation of phenethyl group and glucuronidation (M6), hydroxylation of phenethyl structure (M7), hydroxylation of piperidine ring (M8), ketone formation of phenethyl linker (M9), *N*-oxidation of piperidine and hydroxylation of phenethyl group (M10), *N*-oxidation of 4′-nitrogen (M11) and finally *N*-oxidation of piperidine (M12). The most abundant metabolites were the phase I metabolites of piperidine ring hydroxylation (M8) and *N*-dealkylation (M2), followed by ketone formation of phenethyl linker (M9), *N*-propanoic hydroxylation (M5), phenethyl ring hydroxylation (M7), piperidine *N*-oxidation (M12) and 4′-position nitrogen *N*-oxidation (M11). The only phase II metabolite identified was a glucuronide conjugate of hydroxylated carfentanil (M6). The chemical structures of the identified metabolites are presented in Fig. 4 [128].

### Pharmacology and toxicology

Carfentanil was pharmacologically studied for the first time in 1979 by a scientist group of Janssen Pharmaceuticals, who synthesized it. Its ED_50_ value was determined via a rat hot tail withdrawal test and found to be about 10,000 and 100 times more potent than morphine and fentanyl, respectively [22]. Since then it has been studied extensively among other opioids, but it was never approved for clinical use in humans [23, 28–39]. Carfentanil is mainly used as an anesthetic agent in large animals [45].

Several studies have been conducted for the elucidation of the action of carfentanil through opioid receptors [30–36]. In 1993, [^3^H] carfentanil and [^3^H] [d-Ala^2^-MePhe^4^-Gly-ol^5^]enkephalin (DAMGO) were used as radioligands in order to image high-affinity binding sites in sections of the rat brain. For that purpose 30 brain sections were examined in which absolute and relative densities of high affinity carfentanil binding sites were measured and the autoradiographic image was obtained in order to determine the distribution pattern of the two substances. The highest levels of binding were observed in the striatum section and the lowest levels of binding were observed in the cerebellum. The autoradiographic images showed close distribution patterns for [^3^H] carfentanil and [^3^H] DAMGO, but a remarkable difference in the interaction of the substances with mOR was observed. It is not clear whether the different sensitivity, between [^3^H] carfentanil and [^3^H] DAMGO, in the proteins of the mOR can be attributed to the structural differences [30].

[^11^C] carfentanil and [^11^C] diprenorphine were also used as radioligands in a study measuring the total binding capacity to the opioid receptors, in human volunteers, before and after the administration of naloxone. Twenty-eight volunteers participated in the study. Twenty-one received [^11^C] carfentanil, three [^11^C] diprenorphine, and four received both radioligands. A simple dual detector coincidence system was used for the measurements of the two ligands in the brain [31]. [^11^C] carfentanil was synthesized according to a previously described method [104]. The total binding of [^11^C] diprenorphine was found greater than [^11^C] carfentanil’s, possibly due to either easier transportation of the former through the blood-brain barrier or more binding sites of it in the brain [31].

In a study conducted by Jewett et al. [32], eight derivatives of [^11^C] carfentanil were evaluated as potential mOR agonists within the research for substances with better pharmacodynamics than the parent one. Derivatives were prepared via substitution of aryl- or alkyl-group on a [^11^C]-labeled form of carfentanil and were evaluated for their mOR binding capacity and their pharmacokinetics in mouse brain. Another group of 2-chloro, 2-methoxy and 2-methyl derivatives was also evaluated for their binding within specific brain regions and for their distribution by using an equilibrium infusion rat model [32]. All the [^11^C] derivatives were prepared through *O*-[^11^C] methylation [105] of the corresponding free carboxylic acid [32].

Weltrowska et al. [35] studied the binding affinity of the two isomers of the “carba-analogue” of carfentanil (c-carfentanil) with the opioid receptors. They replaced the nitrogen in the piperidine ring of carfentanil with a carbon in order to assess how the electrostatic interaction of nitrogen with Asp protein of the opioid receptor affects binding and activation of opioid receptors. The *trans* isomer of c-carfentanil was found to result in a 4000-fold decrease in mOR binding affinity than carfentanil, but was still significant. Its δ opioid receptor (dOR) binding affinity was found to be similar to that of mOR, but no receptor selectivity was observed between mOR and dOR receptors. This isomer did not show any significant binding affinity with the κ opioid receptor (kOR). The *cis* isomer did not show any significant binding affinity with any one of the three receptors [35].

Labeled [^11^C] carfentanil has been used in some pharmacological studies as a positron emission tomography (PET) scan radiotracer [33, 34, 39]. More specifically, [^11^C] carfentanil was used in a study for evaluating the response of cocaine users to mOR agonists, including the respective adverse effects. The initial hypothesis was that chronic cocaine exposure might lead to increased brain mOR binding potential. As a result, chronic cocaine users might have a different response to mOR agonists. Carfentanil’s plasma half-life was calculated and found to be 51.4 (±16.2) and 41.8 (±17.5) min for cocaine users and non-drug using controls, respectively. As there were not any significant differences in its half-life among the two groups, they concluded that these differences are most likely pharmacodynamically based. Furthermore, cocaine users were found to have fewer adverse effects (nausea, dizziness, headache, vomiting, and itchiness) than the control ones [33]. [^11^C] carfentanil was again used as a radiotracer in a PET study for the measurement of endogenous opioid release in the brain during painful proximal gastric balloon distension. The volunteers were chosen based on specific criteria as history of drug abuse or psychiatric and gastrointestinal disorders. The study showed up that no endogenous opioid release was detected in the brain during sustained visceral pain and concluded that endogenous opioid levels are more associated with somatic pain [34]. [^11^C] carfentanil was also used as a radioligand in a PET study, in which the mOR availability was measured in the psychiatric disorder of pathological gambling. The results provided an evidence that dysregulation of endogenous opioids might have an important role in the pathophysiology of gambling addiction [39].

Carfentanil is used extensively for immobilizing and tranquillizing mostly large wild animals. Because of this, several animal studies have been conducted in order to clarify the effects of carfentanil on different animal species [45]. Carfentanil was initially evaluated as an anesthetic in guinea pigs, combined with etomidate. Its performance was investigated upon 20 animals of this species weighting from 600 to 800 g. A solution of both substances was prepared and was administrated intramuscularly into the hind leg. Eight of the animals underwent hemodynamic monitoring as well. All the observations showed that this combination of carfentanil and etomidate was sufficient in producing anesthesia and did not influence the circulation and respiration of the animals in dangerous levels. The increased heart rate was attributed to the excitement of the animals, and the recovery period from this drug combination was 90 min [23].

In some other studies ten female and six male captive-born dama gazelles were administrated carfentanil in order to assess the cardiovascular response to it. The drug was administered intramuscularly at doses of 18.4 ± 2.2 μg/kg, and no food was given for 24 h before recumbency. A significant decrease in heart rate, beginning from 5 min after immobilization, was observed. Fifteen minutes after anesthesia was induced, a decrease in respiratory rate became apparent, while hypertension was present over the whole time. Analysis of the arterial blood samples showed that Pa_CO2_ and Pa_O2_ were within normal limits [28].

Carfentanil is the 4-carbomethoxy derivative of fentanyl. It is of great interest that the 4-carbomethoxy derivatization gives as much as 100-fold enhancement of potency of fentanyl as mOR agonist. Vučković et al. [37] synthesized other regioisomers of carfentanil i.e., (±) *cis* and (±) *trans* 3-carbomethoxy fentanyls, and tested their potency as a function of antinociceptive action in rats. As results, the study revealed that the introduction of the 3-carbomethoxy group in the piperidine structure of fentanyl shortened the duration of its action and reduced its potency. It was clarified that the carbomethoxy derivatization at the carbon in 4-position of the piperidine ring is essential to gain such high potency of about 100 times higher than that of fentanyl. However, they suggested that carfentanil regioisomers have to be further evaluated as antinociceptive compounds within the frame of structure-activity relationship studies [37].

Eight domestic goats were used for the clarification of the pharmacokinetic profile of carfentanil and naltrexone by Mutlow et al. [29]. The animals were administrated with 40 μg/kg carfentanil intramuscularly, and after 30 min naltrexone (100 mg naltrexone/mg carfentanil) was administered for anesthesia reversal in several routes of administration (intravenous, intramuscular, and subcutaneous). Blood samples were collected before and after carfentanil administration at different times up to 5 days. Hemodynamic responses were monitored though the entire procedure and also during the collection of the blood samples. The pharmacokinetic profiling of plasma showed rapid carfentanil absorption and a rapid reversal of immobilization by naltrexone after all routes of administration. Carfentanil’s half-life (5.5 h) did not differ according to different administration routes [29].

Naloxone and naltrexone are opioid receptor antagonists and had been used in several cases for the reversal of the respiratory depression caused by carfentanil [29, 109, 129]. Naltrexone was evaluated for antagonizing carfentanil in mOR binding by Miller et al. [109] in an animal study conducted in captive Rocky Mountain elk. Considering the mean immobilization induction time, the mean recovery time and the observed adverse effects, the study concluded that a naltrexone dose of 100 mg/mg of carfentanil is quite effective in reversing carfentanil’s immobilization effects [109]. In another PET scan study conducted by Saccone et al. [129], naloxone was evaluated for its ability to displace [^11^C] carfentanil in the mOR after intranasal and intravenous administration. PET imaging showed that intravenous and intranasal naloxone produced similar decrease in mOR occupancy caused by [^11^C] carfentanil [129]. However, naloxone has a shorter duration of action than that of carfentanil, and multiple doses may be needed to reverse carfentanil’s effects [38, 42, 99, 111, 113, 117, 130]. One or two doses of naloxone are considered enough to treat a heroin overdose. In the case of carfentanil, six or maybe more doses are needed. The DEA suggested the continuous administration of naloxone until the individual’s breathing is resumed for at least 15 min or until emergency medical care arrives [19, 117]. On the other hand, the efficacy of naltrexone in reaching mOR is not as good as that of carfentanil; there is a need to find new opioid antagonists with fewer disadvantages. Yong et al. [38] investigated the efficacy of different doses of nalmefene for antagonizing carfentanil-induced loss of righting reflex and respiratory depression using naloxone as a control in rats. Respiratory parameters and parameters of arterial blood gases were monitored through the procedure. Nalmefene was found to dose-dependently decrease the duration of loss of righting reflex and reverse the respiratory depression caused by carfentanil [38].

Carfentanil is very potent and its effects in humans appear rapidly [43]. These effects include symptoms and signs very similar to those of an opioid intoxication, like cold and clammy skin, nausea, vomiting, pinpoint pupils, disorientation, dizziness, lethargy, sedation, sudden drowsiness, respiratory disorders, possible heart failure, and weak pulse. The DEA advised that anyone experiencing any of the mentioned symptoms should immediately seek medical care [42, 43, 117]. The mentioned symptoms are dose dependent. High doses can lead to severe intoxications and consequently to death [44, 46, 119, 120].

Most adverse effects that carfentanil causes originate from its mOR binding [36]. Mosberg et al. [131] suggested that one way to overcome such adverse effects is by synthesizing compounds with mixed mOR agonist and dOR antagonist properties [131]. Purington et al. [132] had suggested in 2009 that such compounds are certain peptides that had proven to display such properties. In 2015, Váradi et al. [36] conducted a study in which they synthesized ten carfentanil amide analogues and assessed if these analogues could provide analgesia with fewer adverse effects. All of these amides displayed high affinity to the mOR binding, while one displayed high affinity to dOR and low affinity to kOR. This compound was found to provide moderate analgesic efficacy in vivo with no signs of physical dependence and less respiratory depression than morphine [36].

### Intoxications and fatal cases

Carfentanil is a very toxic fentanyl analogue. In veterinary medicine the drug is used as a tranquilizer agent for large animals, but has no proven medical use in humans. It has lately entered the illicit drug market and is responsible for many intoxication cases and deaths all over the world. Since it was first introduced as a chemical weapon at a theater in Moscow, it has been a part of the recently emerged fentanyl analogue crisis. Carfentanil has led to a significant number of deaths, mostly in the United States, and also in Europe [40, 44–46].

The first fatal cases possibly related to carfentanil were reported in Russia, in 2002, when more than 120 people died in the Moscow Dubrovka Theater, in which they were held as hostages by Chechen rebels. During a rescue attempt, the Russian military forces released a “poison” gas though the ventilation system in order to subdue the rebels. Hundreds of hostages were submitted to hospitals suffering from “sleeping gas” poisoning. In the hospital, doctors took several hours testing various antidotes, and after 4 days, the mysterious gas was identified as a fentanyl analogue. Despite the fact that the Russian Health Minister announced that the drug used cannot be characterized as fatal, collectively 127 of the 800 hostages died and 650 required hospital monitoring. The deaths were initially attributed to bad captivity conditions. Evidence suggested that the mysterious “sleeping gas” contained carfentanil and an anesthetic agent like halothane [40]. An LC–MS/MS analysis of clothing and biological fluids from three survivors of the siege revealed the presence of carfentanil along with remifentanil on a survivor's shirt, and a metabolite identified as norcarfentanil was found in a urine sample [41].

Later, in 2013, a series of carfentanil-related deaths was reported in Latvia, and a related alert was issued by the Latvian National Focal Point [44].

In the United States, the first reported carfentanil-related intoxication took place in 2010 in Chicago. It was an unintentional intoxication where a veterinarian was accidentally exposed to carfentanil. A 42-year-old man was anesthetizing elk in order to test them for tuberculosis by using shooting darts containing 1.5 mg of carfentanil citrate and 50 mg of xylazine. When he tried to dislodge a dart, the substance was accidentally splashed into his eyes, face, and mouth. Although he immediately washed his face, he began to feel drowsiness 2 min after. He was at once administered parenterally 100 mg naloxone by his coworkers and was transferred to the medical care unit. On his arrival to the medical center, he only complained of mild and transient chest pain, and his vital signs were evaluated. The size of his pupils and his heart, lung, neurological, and abdominal examinations were within normal limits. He was monitored for 24 h and when stabilized, he was discharged. That was the first reported carfentanil intoxication internationally [45].

In September 2016, the DEA issued an alert informing the police and the public about the dangers of carfentanil. According to the DEA, local law enforcement, and first responders, several overdose deaths were linked to the drug in many parts of the country [120]. Because the DEA published the report on carfentanil, several intoxication deaths have been recorded throughout the states of Florida, Illinois, Colorado, Wisconsin, Minnesota, Michigan, West Virginia, New Hampshire, Virginia, and Maryland. In other states, more unconfirmed cases have been reported [119].

In Ohio, more than 4000 deaths linked to opioids appeared in 2016. In particular, a 36% increase from the previous year was reported. This increase was attributed to heroin and carfentanil abuse. For example, in Akron’s Summit County, nearly half of its 308 overdose deaths were attributed to carfentanil intoxication [133]. In Hamilton County, Ohio, law enforcement agencies recorded 50–70 intoxication cases per week in early 2016. When carfentanil appeared, the number increased to 175–200 per week [119].

In 2017, carfentanil was confirmed as the cause of death in two intoxication cases in Florida. In the first case, a 34-year-old man was found dead in the driver’s seat of a van. A syringe, spoon, and a yellow bag containing a brown powder were found in the cup holder near the seat. He had a history of tobacco, alcohol, marijuana, and heroin abuse, and mild hypertensive heart disease and a mild hepatic steatosis were found at autopsy. Toxicological analysis revealed the presence of carfentanil in heart blood along with furanylfentanyl, fentanyl, morphine, and hydromorphone at concentrations 1.3 ng/mL for carfentanil, 0.34 ng/mL for furanylfentanyl, 6 ng/mL for fentanyl, and <20 ng/mL for both morphine and hydromorphone. Additionally, 6-acetylmorphine along with hydrocodone and hydromorphine was present in his vitreous humor, and morphine, hydromorphone, 6-acetylmorphine, hydrocodone and hydromorphine were present in his urine. The cause of death was pronounced as intoxication due to heroin, fentanyl, carfentanil, and furanylfentanyl. In the second case, a 25-year-old man was found unconscious on his mattress by his mother in a tent where he was living. The mother called emergency services, and on arrival of medical responders, he was pronounced dead. A bag with a brown powder was found next to the deceased. The toxicological analysis showed a concentration 0.12 ng/mL of carfentanil in heart blood, 460 ng/mL of benzoylecgonine in peripheral blood, 510 ng/mL of benzoylecgonine, and 40 ng/mL of cocaine in the vitreous humor. The medical examiner declared accidental carfentanil intoxication as the cause of death [46].

Several other reports concerning fatal cases involving carfentanil can be found on web sources. All these cases occurred in the United States [134–141].

### Analysis of carfentanil in seized materials and biological specimens

Many analytical methods for determining carfentanil in seized materials and biological specimens have been described through the years. Some have been specifically developed for this purpose, while others within the frame of the investigation of specific carfentanil-related intoxication cases [26, 41, 46, 90, 101, 128, 142–144].

An ^125^I-radioimmunoassay (^125^I-RIA) detection method was published in 1989, in which the ability of sevn antibodies to fentanyl derivatives developed to react with carfentanil was investigated. The seven antibodies were evaluated in vivo for their binding ability with fentanyl, carfentanil, and four other analogues. The ability was evaluated by measuring the concentration of carfentanil that is required to reduce maximum binding to 50%. Carfentanil cross-reacted well with only one antibody, while less satisfactory cross-reactivity was observed with the six other antibodies [26].

Tobin et al. [142] developed and evaluated a one-step ELISA test for sufentanil, which also can cross-react with carfentanil and detect it in horse urine several hours after its administration [142]. Carfentanil is included in a screening test based on ELISA principles that were developed recently by Randox Laboratories Ltd. for “Designed fentanyl and opioids” along with other fentanyl analogues in an NPS panel as described before in this review [90].

Hunter et al. [143] developed and validated the first chromatographic method for determining carfentanil and naltrexone in goat plasma. Plasma samples were extracted two times with toluene after pretreatment with 1 M NaOH. The extracts were dried under N_2_ in a warm water bath and injected into the LC–MS chromatographic system. The analysis was isocratically performed with acetonitrile/(10 mM ammonium acetate and 0.1 mM citrate) (30:70, v/v). A Zirchrom PBD column was used and the flow rate was set in 0.3 mL/min. The mass spectrometer was set at a single ion monitoring mode. The lower limit of quantification was 8.5 pg/mL for carfentanil and 0.21 ng/mL for naltrexone [143].

Another chromatographic method was developed and validated by Wang et al. [144] to analyze carfentanil and norcarfentanil along with 11 other fentanyl analogues in human urine. All urine samples were extracted through SPE Oasis HLB^®^ C18 columns. Initially, 0.5-mL urine samples were spiked with an IS mixture of deuterated analytes including carfentanil-*d*_5_ and norcarfentanil-*d*_5_ and acetate buffer (pH 4.0). The samples were then extracted via SPE columns, concentrated and transferred in autosampler microvials. The eluents were dried, reconstituted in water and injected into the LC–MS/MS chromatographic system. The chromatographic column was a Waters Xterra MS C18 column and the system was operated in a gradient mode with a flow rate 0.5 mL/min. Mobile phase A was ammonium acetate in HPLC water and mobile phase B was ammonium acetate in acetonitrile/methanol (95:5, v/v). The method was validated and the limit of detection (LOD) for each analyte was determined. The LODs for carfentanil and norcarfentanil were found to be 0.003 and 0.027 ng/mL, respectively [144].

An LC–MS/MS method was used for the analysis of the clothing extracts of two survivors of the Moscow theater siege, and urine from a third survivor. A jumper and a leather jacket were collected from one victim, a shirt from another, and two blood samples from each. A single urine sample was obtained from the third survivor. Clothing samples and blood samples were initially screened by a GC–MS system and a GC–MS/MS system, respectively, for fentanyl, *cis*-3-methylfentanyl, carfentanil, sufentanyl, lofentanil, and remifentanil. Clothing samples were extracted with solvent (CH_2_Cl_2_) or water and blood samples were liquid–liquid extracted with 1-chlorobutane prior to screening. Results after analysis of clothing and blood samples were negative for these substances. All clothing samples and the urine from the third victim were further cleaned with SPE according to a previously published method by Shou et al. [145] prior to LC–MS/MS analysis. The SPE columns were preconditioned with methanol/water/5% acetic acid solution and after that the cartridges were washed with 5% aqueous acetic acid and methanol. Subsequently, they were eluted twice with 2% aqueous NH_4_OH in chloroform/isopropanol (4:1, v/v). The eluents were dried, reconstituted with acetonitrile/water/trifluoroacetic acid (TFA) (95:5:0.05, v/v/v) and injected to the chromatographic system. The system performed in isocratic mode with 7% of 0.05% TFA in water/93% of 0.05% TFA in acetonitrile. A Betasil Silica-100 chromatographic column was used at a flow rate of 200 μL/min [41].

Feasel et al. [128] used both low-resolution and high–resolution mass spectrometry systems for the assessment of carfentanil’s metabolic clearance and for the determination of in vitro carfentanil’s metabolites, respectively. For the low-resolution HPLC–MS/MS, the samples were injected into the HPLC chromatographic system, which was set in gradient mode with 0.1% formic acid in water as phase A and 0.1% formic acid in acetonitrile as phase B. A Kinetex™ C18 chromatographic column was used and the flow rate was set at 0.5 mL/min. For the identification of possible metabolites, an HPLC–triple TOF analysis was performed under the same chromatographic conditions (column and mobile phases) as with the HLM analysis [128].

Seized powders from three exhibits were subjected to profiling analysis via infrared spectroscopy, nuclear magnetic resonance spectroscopy, GC–MS, and isotope ratio mass spectrometry. Spectral data for carfentanil citrate and carfentanil hydrochloride were provided in the study. The quantification of carfentanil was performed by GC combined with flame ionization detection. For the GC–MS analysis, CH_2_Cl_2_ extract of carfentanil was injected via split mode into the system with a flow rate at 36.5 cm/s of helium. A DB-1 fused silica capillary column was used. Fentanyl, furanylfentanyl, acetylcarfentanil, heroin, 6-acetylmorphine, acetylcodeine, noscapine, and diphenhydramine were detected in the seized materials along with carfentanil [101].

A GC–MS screening method was used for the determination of carfentanil in two fatal intoxication cases involving carfentanil and furanylfentanyl. The urine and blood samples were pretreated with alkaline borate buffer and a mixture of toluene/hexane/isoamyl alcohol (78:80:2, v/v/v). Subsequently, the samples were back extracted into ethyl acetate using sulfuric acid and neutralization with NaHCO_3_/K_2_CO_3_. An Rtx-5 column was used for the chromatographic analysis. Case 1 had carfentanil and furanylfentanyl at concentrations of 1.3 and 0.34 ng/mL in blood, respectively. Case 2 had a carfentanil concentration at 0.12 ng/mL in blood [46].

### Legal status

Carfentanil is one of the most dangerous fentanyl analogues to come into the drug market in recent years. As a result, there is an undeniable need for the safety of the public for it to be regulated in order to minimize the potential of an imminent health hazard as much as possible. The drug is not scheduled as of now under the 1961 United Nations Convention, but it is a controlled substance in many countries in Europe, as well as the United States, Canada, Australia, and China [121, 146–157].

In Czech Republic, carfentanil is regulated under the Government regulation concerning lists of drugs issued on December 18, 2013 [147]. Carfentanil was included in Annex 1 of Danish Executive Order No.557 of 31 May 2011, which came in force on November 24, 2016; the drug was included on the B List, and it can be used only for medical and scientific purposes [148]. In the United Kingdom carfentanil is controlled under the Misuse of Drugs Act 1971 [149].

In the United States, the DEA classifies carfentanil as a Schedule II substance according to the Federal Controlled Substances Act issued on October 28, 1988 [150–152]. On September 29, 2016 the Department of Health in Pennsylvania issued the placement of carfentanil in Schedule II under the Controlled Substance, Drug, Device, and Cosmetic Act [150].

In Canada, carfentanil is a Schedule I substance under Canada’s Controlled Drugs and Substances Act, which was last amended on May 18, 2017 [153, 157].

In Australia, carfentanil has been included in the Poisons Standard in June 2017, by the Australian Department of Health. It has been clarified as a schedule 8 poison, and legal restrictions apply to its manufacture, supply, distribution, possession, and any type of abuse, physiological misuse, or physical dependence [154].

China started to discuss scheduling carfentanil 4 months after the Associated Press discovered 12 Chinese laboratories willing to export carfentanil to the United States, Canada, and Europe in October 2016. The scheduling came into force in March 2017 when it was added to the list regulated by the “Administrative Measures on Narcotics and Psychotropic Substances without Medical Use” [121, 155, 156].

## Conclusions

The fentanyl problem is constantly growing. New fentanyl analogues continuously invade the drug arena as dealers and traffickers try to stay ahead of the law. Two such analogues are ocfentanil and carfentanil. Ocfentanil was synthesized in 1986, while carfentanil was synthesized earlier in 1974. Nevertheless, they only recently appeared on the drug market and are a part of the recent opioid epidemic. Today, they are mostly manufactured in China and are distributed from there to many parts of the world. There is evidence indicating that these opioids are misused or abused worldwide, mainly in Europe and the United States. Conversations taking place on drug forums show that these fentanyl analogues are already well-known among drug addicts. Their presence on the drug market is also pointed out in reported ocfentanil- and carfentanil-related intoxication cases and deaths, web data referring to their illicit trafficking or drug forum conversations, as well as by the fact that they are regulated or being considered for regulation in many parts of the world. Ocfentanil and carfentanil are undeniably very dangerous opioid drugs and a very serious matter of concern when it comes to public safety. Authorities should take appropriate actions in order to avoid the expansion of this threat by taking proper and prompt measures.
